# uORF Shuffling Fine-Tunes Gene Expression at a Deep Level of the Process

**DOI:** 10.3390/plants9050608

**Published:** 2020-05-11

**Authors:** Yukio Kurihara

**Affiliations:** Synthetic Genomics Research Group, RIKEN Center for Sustainable Resource Science, 1-7-22 Suehirocho, Tsurumi-ku, Yokohama, Kanagawa 230-0045, Japan; yukio.kurihara@riken.jp

**Keywords:** uORF, translation, ribosome, NMD

## Abstract

Upstream open reading frames (uORFs) are present in the 5’ leader sequences (or 5’ untranslated regions) upstream of the protein-coding main ORFs (mORFs) in eukaryotic polycistronic mRNA. It is well known that a uORF negatively affects translation of the mORF. Emerging ribosome profiling approaches have revealed that uORFs themselves, as well as downstream mORFs, can be translated. However, it has also been revealed that plants can fine-tune gene expression by modulating uORF-mediated regulation in some situations. This article reviews several proposed mechanisms that enable genes to escape from uORF-mediated negative regulation and gives insight into the application of uORF-mediated regulation for precisely controlling gene expression.

## 1. Introduction

In the 5’ untranslated regions (UTRs) of mRNAs, there are a variety of cis-regulatory sequence elements that affect the subsequent fate (e.g., transportation, stability and translation efficiency), of the mRNAs [1]. One of the most characterized elements is an upstream open reading frame (uORF) that is distinct from the protein-coding main ORF (mORF) on the same polycistronic mRNA [2]. It is estimated that up to half of genes possess one or more uORFs in *Arabidopsis thaliana* [2]. Emerging ribosome profiling approaches have revealed that uORFs themselves can possibly be translated into short peptides [3,4,5,6,7,8,9] and that translation of some uORFs starts with non-AUG initiation codons [10,11]. Peptide lengths encoded by uORFs are variable but are typically between 1–100 amino acids [12]. Taking into consideration the actual translation of uORFs, it is better to rephrase “5’UTR” as “5’ leader sequence” in this review.

**Figure 1:**
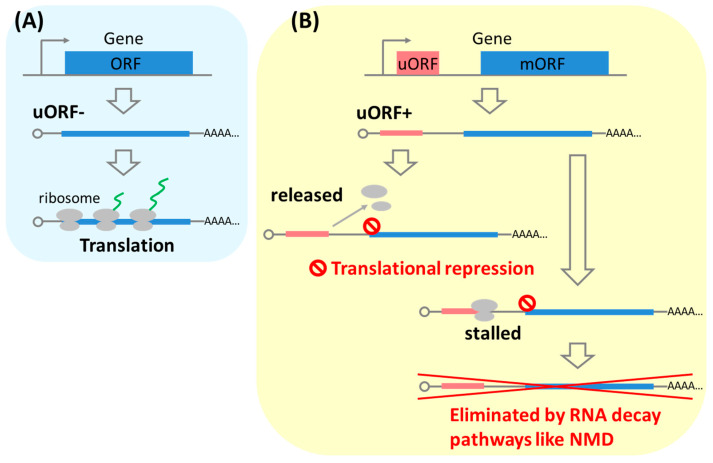
Overview of upstream open reading frame (uORF)-mediated repression of gene expression. (**A**) The uORF mRNA is translated into protein by ribosomes. (**B**) On the other hand, uORF-mediated repression of the main ORF (mORF) occurs in polycistronic uORF+ mRNA. Translation repression of an mORF occurs by ribosome releasing after uORF translation or ribosome stalling in the uORF. Some are eliminated by RNA decay pathways like nonsense-mediated mRNA decay (NMD).

It is well known that uORFs negatively affect expression of downstream protein-coding mORFs [2,13,14]. In this context, uORFs act as repressors of translation of mORFs (Figure 1). The general process of eukaryotic translation is well described in previous review articles [6,15]. uORF translation precedes mORF translation, because ribosome scanning starts from the cap structure at the 5’-distal end. It is proposed that uORF-mediated translational repression of mORFs occurs by releasing ribosomes after uORF translation or by stalling ribosomes in the uORF and then preventing them from accessing the mORF [2]. Some features, such as an increasing number of uORFs and a shorter distance between the uORF and mORF, may enhance efficiency of uORF-mediated repression of mORF translation [16]. A recent paper revealed that even minimum uORFs comprising AUG-STOP inhibit translation of mORFs by stalling ribosomes on the uORFs in *Arabidopsis* [17]. It is known that conservation in the sequence of uORF-encoded peptides among plant species is an important factor for the efficiency of uORF-mediated translational repression, because mutations reducing the conservation diminished the repressive efficiency of the uORFs [18,19]. Furthermore, some physiological roles of plant-conserved uORFs have been reported [20].

In addition, uORFs can also act as triggers of nonsense-mediated mRNA decay (NMD), which is a selective mRNA decay mechanism (Figure 1) [21,22]. In this case, it has been speculated that the stop codon of the uORF and the long provisional 3’UTR—which starts downstream of the uORF—are recognized as NMD targets [23] and the mRNA is thus selectively degraded. Transcripts with longer uORFs are likely to be more efficiently eliminated by NMD in plants [24]. Uchiyama-Kadokura et al. suggest that induction of NMD by the uORF of the *AdoMetDC1* mRNA is associated with ribosome stalling at the uORF stop codon in *Arabidopsis*. A similar relationship has been reported in yeast, supporting the possibility that ribosome stalling at a uORF could be one of the determinants of NMD [25,26]. In other cases, ribosome stalling might induce other RNA decay like no-go decay [27]. Thus, uORFs repress gene expression not only as translational repressors but also as triggers of RNA decay.

## 2. Mechanisms for Evading uORF-Mediated Regulation

Some mechanisms for evading uORF-mediated negative regulation of gene expression have been proposed. This review explains not only well-known ones (leaky scanning and reinitiation) but also more recent ones, including those that have been experimentally proven and those that have yet to be. This includes alternative transcription start sites (TSSs) selection, splicing out of uORF initiation codons and initiation of cap-independent translation. In particular, alternative TSS and splicing can produce two mRNA variants, uORF+ and uORF–. Some plant stimuli result in shuffling the amount of variants by inducing a TSS positional shift or alternative splicing. 

### 2.1. Leaky Scanning and Reinitiation 

Viruses often use leaky scanning by ribosomes and translational reinitiation to produce more than one kind of protein from their polycistronic RNAs [28]. Eukaryotes, including plants, also use leaky scanning and reinitiation to evade uORF-mediated translational repression and then translate mORFs. Their mechanisms have been analyzed often and reviewed by others [20,29], so they are introduced only briefly here. 

**Figure 2:**
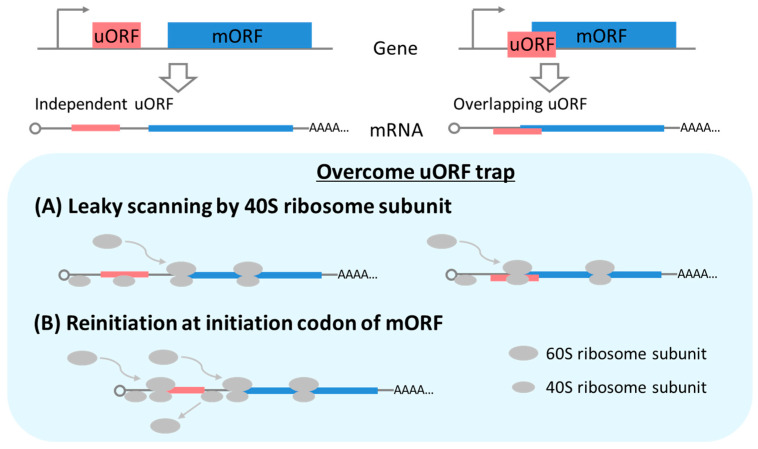
Schematic representation of leaky scanning (**A**) and translational reinitiation (**B**). There are two types of uORFs. One is an independent uORF that does not overlap with an mORF and the other is a uORF that does overlap. Two mechanisms, leaky scanning and translational reinitiation, can overcome the uORF-mediated repression of an mORF.

Translation generally starts by recruiting 40S ribosome subunits to the 5’ cap structures of the mRNAs. In canonical scanning, uORF is first recognized prior to the mORF. However, in leaky scanning, the 40S scanning ribosomal subunit in 48S preinitiation complexes passes over the initiation codon of the uORF avoiding uORF translation (Figure 2A). When the 40S scanning subunit reaches the initiation codon of the mORF, the 60S ribosomal subunit is recruited, forming an 80S-translating ribosome with a 40S subunit. Once this happens, translation of the mORF starts. 

In another case, after translation of a uORF, the translating ribosome is disassembled and the 60S subunit is released (Figure 1B). In translational reinitiation, the remaining 40S subunit on the mRNA still continues scanning the downstream initiation codon and an 80S-translating ribosome is formed again at the initiation codon of the mORF (Figure 2B). 

Some events of leaky scanning or translational reinitiation have been reported in *Arabidopsis* [20]. For example, ribosomes are stalled at the stop codon of the second uORF of *bZIP11* mRNAs in a peptide sequence-dependent manner under high sucrose conditions, resulting in low translation of a bZIP-encoding mORF [30,31]. This translational repression of an mORF is alleviated by sucrose depletion because leaky scanning may occur. Thus, environmental changes sometimes reinforce translational initiation of mORFs. Leaky scanning of uORFs and reinitiation of the mORF on the *GCN4* mRNA in yeast have also been well exemplified [15,32]. Taking this and other evidence into account [2], translational reinitiation or leaky scanning may be the most common mechanism for evading uORF-mediated translational repression of mORFs.

There are two types of uORF: one is an independent uORF that does not overlap with the mORF and the other is an overlapping uORF (Figure 2A). Leaky scanning but not reinitiation can overcome overlapping uORF-mediated repression.

### 2.2. Selection of Transcription Start Sites

Approximately 75% of genes are estimated to use multiple TSSs in *Arabidopsis* [33,34]. The TSS is one of the most striking determinants of the 5’ leader sequence. It is conceivable that the leader sequences of transcripts derived from more upstream TSSs possess more information than those from downstream TSSs. In this context, a few papers have reported that the selection of alternative TSSs affects the translation of genes [33,35].

**Figure 3:**
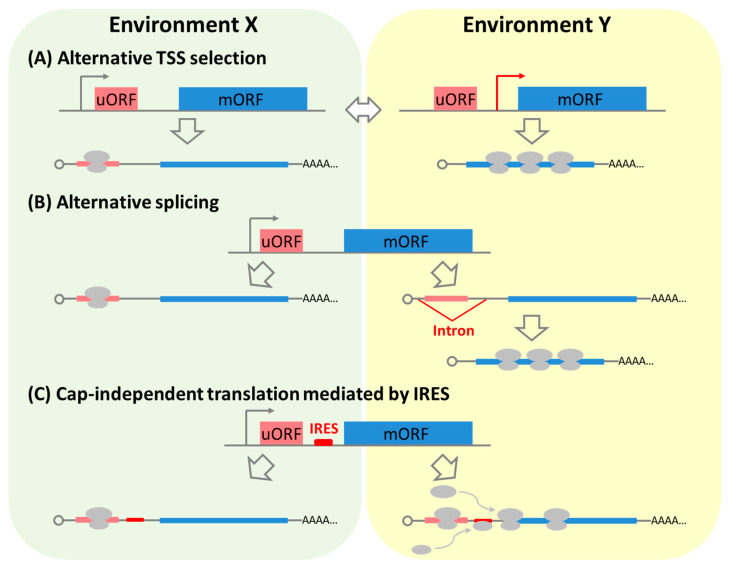
Schematic representation of three newly proposed mechanisms for evading uORF-mediated repression of gene expression. (**A**) Alternative transcription start site (TSS) selection. (**B**) Alternative splicing out of the uORF. (**C**) Cap-independent translational initiation mediated by an internal ribosomal entry site (IRES). The environment around plants may affect the occurrence of these mechanisms.

Transcription from TSSs upstream of the uORF (uTSS) produces uORF+ mRNAs, while that from downstream TSSs (dTSSs) generates uORF– mRNAs [33,36]. It is assumed that environmental differences may reinforce the occurrence of alternative TSS selection, which determines whether a gene dominantly produces uORF+ or uORF– mRNAs. In fact, our previous report showed that the main TSS position in more than two hundred light-responsive genes was shifted from upstream of the uORF to downstream of the translation initiation codon of the uORF (Figure 3A), when young *Arabidopsis* seedlings grown in dark were exposed to blue light [33]. Ribosome profiling analysis demonstrated that these TSS shifts were likely to upregulate translation efficiency of mORFs upon blue light exposure, indicating that blue light alleviated uORF-mediated translational repression in genes by positionally shifting the main TSS, resulting in the production of more uORF– mRNAs. Furthermore, in some of the genes showing a TSS shift, accumulation of uORF+ mRNAs was detected in *upf1* NMD-deficient mutants compared to wild-type plants, indicating they are targeted by NMD [33]. Taken together, these TSS shifts from uTSS to dTSS not only avoid uORF-mediated translational repression of the mORFs but also NMD in some genes.

Alternative TSS selection as described above can also overcome translational repression by uORFs overlapping with mORF initiation codons.

### 2.3. Alternative Slicing Excluding or Modifying uORFs

In addition to alternative TSSs, alternative splicing is also one of the most important determinants of the 5’ leader sequence. Indeed, it has been reported that alternative splicing that occurs in the 5’ leader sequence also affects the translation efficiency of genes in plants [35].

Splicing out of introns containing the initiation codon of the uORF or a part of the uORF sequence from the 5’ leader sequence of pre-mRNAs produces mRNAs without uORFs or mRNAs with truncated uORFs, respectively (Figure 3B), while intron retention produces mRNAs with full uORFs. Furthermore, the splicing out of an intron located between the uORF and the mORF reduces the distance between the two. As with alternative TSS selection, such splicing has the potential to avoid NMD and so it is possible that the gene evades or alleviates uORF-mediated repression by splicing out uORF sequences. In contrast, alternative splicing may produce a *de novo* initiation codon of a uORF at the splicing junction. 

However, at present, there have been a limited number of examples showing a possible link between splicing and uORFs [2,37,38,39,40]. For example, Pasentsis et al. showed that the *PHY2* gene in *Ceratodon purpureus* produces two splicing variants harboring different uORF sequences in a light-dependent manner [39]. Combier et al. suggest that, in *Medicago truncatula*, alternative splicing of an intron containing uORFs in the *HAP2-1* mRNA regulates root nodule development [40].

Alternative splicing events frequently occur in response to environmental changes in plants [41]. It is easy to speculate that splicing of uORF-containing introns might be responsible for the plant’s physiological response through fine-tuning the translation of the gene. Therefore, some issues, such as how many genes undergo alternative splicing of uORFs, which stimuli induce them and whether splicing out of the uORF actually impacts translation or expression of genes, remain to be elucidated by future research.

### 2.4. Cap-Independent Translation Initiation

Cap-independent translation is commonly driven by internal ribosomal entry sites (IRESs) that form dynamic RNA secondary structures. IRESs are widely used by viruses for producing proteins from their polycistronic mRNAs [42]. In plant systems, a viral IRES has been used for expressing two genes from a polycistronic mRNA [43,44].

A variety of endogenous IRESs or IRES-like structures have been discovered not only in viruses but also in eukaryotes including plants [45,46,47,48]. If an IRES is located between the initiation codon of a uORF and an mORF, internal entry of ribosomes should overcome uORF-mediated translational repression as proposed in Figure 3C. 

Although there have not been any examples illustrating such avoidance of translational repression (Figure 3C) at least in plants, some work has reported that environmental changes enhance the occurrence of IRES-dependent translation in plants [47,48]. For example, the IRES-mediated translation of the *WUSCHEL mRNA* in *Arabidopsis* is enhanced by environmental hazard stress [47]. In addition, recent reports have demonstrated that *N*^6^-methyladenosine (m^6^A) methylation of 5’ leader sequences promotes cap-independent translation by IRESs in mammals [49,50]. Thus, it is speculated that environmental changes could induce hypermethylation of adenosine in 5’ leader sequences and then promote IRES activity. 

Regarding m^6^A modification, it has also been reported that amino acid starvation induces demethylation at the m^6^A site within overlapping uORF on the mammalian *ATF4* mRNA and increases mORF translation [51]. However, in this case, translation of the *ATF4* mORF may be cap-dependent but not cap-independent.

## 3. Application of uORF-Mediated Regulation

Regulatory mechanisms of gene expression have often been modified and/or applied to expressing preferable genes under particular conditions. Application of transcriptional regulation using tissue-specific or stimulus-inducible promoters is the most popular. Recent papers have reported that translational fine-tuning by artificial modification of uORFs enables proper transgene expression to be achieved [14,52]. In particular, Zhang et al. showed that CRISPR/Cas9 editing of the uORF sequence of the *GGP2* gene encoding a key enzyme for vitamin C biosynthesis results in an increased amount of vitamin C through enhanced mORF translation in lettuce [14].

This review proposes that there are various ways this regulation can be applied. A dynamic range of spatial and temporal gene expression is controlled at the transcriptional level. In the case where an appropriate level of gene expression is required, fine-tuning by translational regulatory elements such as uORFs can be applied to elicit the required function of the gene. Adjustment of gene expression may also be achieved by combining the use of both uORFs and IRESs. In particular, the uORF is an attractive candidate for controlling gene expression at the translational level.

## 4. Future Perspectives 

This review introduces several mechanisms that can overcome uORF-mediated translational repression of mORFs in polycistronic mRNAs. In particular, uORF shuffling by alternative TSS selection or alternative splicing may be a notable concept. It is expected that a growing body of future research will elucidate the unknown parts of the mechanisms. For this purpose, integrated analysis of multiple omics data derived from not only the transcriptome but also ribosome profiling and proteome will be required. As effectiveness of uORFs on mORF translation varies case by case and the action of IRESs is still obscure in plant biological processes, deeper understanding of the regulatory mechanisms of gene expression is necessary for their application in crops.
